# Paracrine Anti-inflammatory Effects of Adipose Tissue-Derived Mesenchymal Stem Cells in Human Monocytes

**DOI:** 10.3389/fphys.2018.00661

**Published:** 2018-05-31

**Authors:** Maria I. Guillén, Julia Platas, María D. Pérez del Caz, Vicente Mirabet, Maria J. Alcaraz

**Affiliations:** ^1^Instituto Interuniversitario de Investigación de Reconocimiento Molecular y Desarrollo Tecnológico (IDM), Universitat Politècnica de València – Universitat de València, Valencia, Spain; ^2^Department of Pharmacy, Faculty of Health Sciences, CEU Cardenal Herrera University, Valencia, Spain; ^3^Department of Plastic Surgery and Burns, Hospital Universitario y Politécnico La Fe, Valencia, Spain; ^4^Valencia Transfusion Center, Valencia, Spain

**Keywords:** mesenchymal stem cells, inflammation, monocytes/macrophages, oxidative stress, inflammatory mediators

## Abstract

The inflammatory process is an essential phenomenon in the induction of immune responses. Monocytes are key effector cells during the inflammatory process. A wide range of evidence indicates that mesenchymal stem cells from adipose tissue (ASC) are endowed with immunomodulatory capacity. However, the interaction between ASC and monocytes in the innate immune response is not well understood. The aim of this work was to investigate the possible paracrine anti-inflammatory effects of ASC in human monocytes. Monocytes were isolated from buffy coats and ASC from fat of non-obese patients. Conditioned medium (CM) from ASC in primary culture was used. We have assessed the effects of CM on the production of inflammatory mediators, degranulation, migration, phagocytic activity, senescence, oxidative stress, mitochondrial membrane potential and macrophage polarization. We have shown that ASC exert paracrine anti-inflammatory actions on human monocytes. CM significantly reduced the production of TNFα, NO and PGE_2_ and the activation of NF-κB. In addition, we observed a significant reduction of degranulation, phagocytic activity and their migratory ability in the presence of the chemokine CCL2. The senescence process and the production of oxidative stress and mitochondrial dysfunction were inhibited by CM which also reduced the production of TNFα by M1 macrophages while enhanced TGFβ1 and IL-10 release by M2 macrophages. This study have demonstrated relevant interactions of ASC with human monocytes and macrophages which are key players of the innate immune response. Our results indicate that ASC secretome mediates the anti-inflammatory actions of these cells. This paracrine mechanism would limit the duration and amplitude of the inflammatory response.

## Introduction

Numerous investigations have demonstrated the high potential of mesenchymal stem cells (MSC) for the development of therapeutic strategies in tissue repair and control of inflammatory conditions (Law and Chaudhuri, 2013). Many reports have also shown that modulation of inflammation may contribute to the beneficial effects of MSC which could depend on the production of soluble factors or cell-cell contact (Sheng et al., 2008; Prockop and Oh, 2012). It is known that MSC exert immunomodulatory effects on innate and adaptive immune systems (reviewed in Law and Chaudhuri, 2013; Tofiño-Vian et al., 2018). The immunomodulatory and anti-inflammatory properties of MSCs have supported studies on cellular therapy for inflammatory autoimmune diseases such as rheumatoid arthritis, systemic lupus erythematosus and systemic sclerosis. In particular, human adipose tissue-derived mesenchymal stem cells (ASC) have demonstrated an interesting immunomodulatory potential (reviewed in Mattar and Bieback, 2015). Recently, we have observed that the *in vivo* anti-inflammatory effects of ASC can be reproduced by the administration of their conditioned medium (CM) in the zymosan-injected air pouch model (Carceller et al., 2015). We have also shown the anti-inflammatory and anti-senescence effects of CM from human ASC in osteoarthritic chondrocytes (Platas et al., 2013, 2016). A better knowledge of ASC paracrine properties may help to develop novel approaches for the treatment of inflammatory conditions.

To gain further insight into the paracrine effects of ASC, we have focused this study on human monocytes and macrophages which play a central role in innate immunity. These cells produce a wide range of inflammatory mediators subjected to regulatory mechanisms. Monocyte activation helps cells to remain viable in inflammatory microenvironments but a resolution failure results in continuous inflammation. Therefore, an exaggerated or prolonged activation leads to self-amplifying stimulation of immune cells and damaging effects on different cell types which are involved in the pathogenesis of chronic inflammatory diseases such as rheumatoid arthritis, inflammatory bowel disease, neurodegenerative disorders, atherosclerosis, etc. (Greaves and Channon, 2002; Parihar et al., 2010). In the present work, we have extended our studies on ASC paracrine effects, by characterizing the regulation of relevant inflammatory responses and major functions of human monocytes and macrophages by CM from ASC.

## Materials and Methods

### Isolation and Culture of Cells

The design of the work was approved by the Institutional Ethical Committees (University of Valencia and La Fe Polytechnic University Hospital, Valencia, Spain). Samples were obtained from donors after they provided informed written consent according to the Helsinki Declaration of 1975, as revised in 2013. Adipose tissue was obtained from healthy non-obese adults who had undergone abdominoplasty (11 women and 2 men, aged 54.1 ± 7.4 years, mean ± SEM). Samples were washed with phosphate-buffered saline (PBS), minced, digested at 37°C for 1 h with 1% of type I collagenase (Gibco, Life Technologies, Madrid, Spain), and filtered through a 100 μm cell strainer (BD Biosciences Durham, NC, United States). Cells were then washed with DMEM/HAM F12 (Sigma-Aldrich, St. Louis, MO, United States) containing penicillin (500 U/ml) and streptomycin (500 U/ml), seeded onto tissue culture flasks (350,000/25 cm^2^) in medium supplemented with 15% human serum from whole-blood donations of AB-blood-group-typed donors according to the criteria of Valencia Transfusion Center (Valencia, Spain), and incubated with 5% CO_2_ at 37°C. When the cells reached the semi-confluence, tissue culture plates were washed to remove any residual non-adherent cells. The phenotype of ASC was analyzed by flow cytometry (FACS-Canto II, BD Biosciences, San Jose, CA, United States) with specific antibodies, anti-CD105-PE, anti-CD90PerCP-eFluo710, anti-CD34APC (eBioscience, Inc., San Diego, CA, United States), and anti-CD45-PE (BD Pharmingen, BD Biosciences), and cellular viability with propidium iodide. More than 98% of viable cells were positive for CD105 and CD90, and negative for CD45 and CD34. CM was collected from cells at passages 0 and 1 at 48 h of culture, pooled, centrifuged, and stored at -80°C in sterile conditions. We performed a cytokine profiling of CM using the RayBio^®^ Human Cytokine Antibody Array C6 (RayBiotech, Norcross, GA, United States) according to manufacturer’s instructions. Detection of chemiluminescence was performed by the AutochemiTM System with the Labworks 4.6 program (UVP Inc., Upland, CA, United States). Image J program (NIH, Bethesda, MD, United States) was used for analysis of results. Compared with control medium, the array revealed in CM elevated signals for interleukin (IL)-6 and IL-10, CXCL6, CCL7, CCL22, and CCL23.

Human monocytes were isolated from buffy coats provided by the blood bank Valencia Transfusion Center. Samples were mixed with DMEM/HAM F12 containing penicillin (500 U/ml) and streptomycin (500 U/ml) in a 1:1 ratio and centrifuged for 15 min at 400 × *g* and 18–20°C. The pellet was resuspended in the above medium and this suspension was added to tubes containing Ficoll-Paque Premium 1.073 (GE Healthcare, Barcelona, Spain) in a slow stream to maintain the gradient. The tube was then centrifuged for 40 min at 400 × *g* and 18–20°C. The top layer was aspirated and the mononuclear cell fraction was collected from the interface. Cells were washed with medium, viability was assessed by the Trypan blue method and then they were seeded at 10^6^/ml in medium supplemented with 10% human serum. After 2 h incubation, cells were washed with medium and adherent cells were characterized by flow cytometry using a FACS-Canto II cytometer (BD Biosciences), anti-CD45-PE (BD Pharmingen, BD Biosciences) and anti-CD14-PE (eBioscience, Inc.) antibodies and propidium iodide. More than 98% of viable cells were positive for CD45 and CD14. To perform the experiments, cells were incubated in medium supplemented with 10% human serum and stimulated with different agents and for different times, as indicated, in the presence or absence of CM (100% of medium, 0.4 ml for 24-well plates, 1 ml for 6-well plates).

### MTT Assay

The mitochondrial reduction of 3-(4,5-dimethylthiazol-2-yl)-2,5 diphenyltetrazolium bromide (MTT) to formazan as an indicator of cell viability was assayed in monocytes treated with CM or medium in the presence or absence of lipopolysaccharide (LPS, from Escherichia coli 0111:B4, Sigma-Aldrich), (1 μg/ml) and incubated at 37°C for 24 or 72 h. MTT (200 μg/ml) was then added and incubation proceeded for 2 h. Medium was removed and cells were solubilized in dimethyl sulfoxide (100 μl) to quantitate formazan at 550 nm using a Victor3 microplate reader (PerkinElmer Spain, Madrid, Spain).

### Determination of TNFα, NO and PGE_2_ Production by Human Monocytes

Monocytes were incubated with CM in the presence or absence of LPS (1 μg/ml) at 37°C for 24 h. Supernatants were used to measure tumor necrosis factor-α (TNFα) by an ELISA assay (Invitrogen, Thermo Fisher Scientific), with sensitivity of 4 pg/ml, nitric oxide (NO) production by fluorometric determination of nitrite levels (Misko et al., 1993) using a Victor3 microplate reader (PerkinElmer Spain), and prostaglandin E_2_ (PGE_2_) by radioimmunoassay (Moroney et al., 1988).

### Myeloperoxidase

Monocytes were incubated with CM and/or 12-O-tetradecanoylphorbol-13-acetate (TPA) (300 nM, Sigma-Aldrich) at 37°C for 3 or 24 h. Supernatants were used to measure myeloperoxidase activity by using 3,3′,5,5′-tetramethylbenzidine (Sigma-Aldrich) as substrate as previously described (De Young et al., 1989). Absorbance at 450 nm was quantified in a Victor3 microplate reader (PerkinElmer Spain).

### Cell Migration

Cell migration was assayed with 8-μm-pore size Transwell migration chambers (Thermo Fisher Scientific) (Bronkhorst et al., 2014). Monocytes (10^6^ cells) in 1 ml of DMEM/Ham’s F-12 with antibiotics and 10% human serum or in 1 ml of CM were added to the upper chamber. In the lower chamber, the chemokine CCL2 (100 ng/ml, Peprotech EC Ltd, London, United Kingdom) was added to the medium. Heparan sulfate proteoglycan could influence the effects of CM on cell migration (Shute, 2012). Therefore, we included experimental groups treated with CM previously incubated with anti-heparan sulfate proteoglycan antibody (Sigma-Aldrich, clone A7L6, 10 μg/ml) for 2 h. Cell migration was allowed to proceed for 72 h at 37°C and 5% CO_2_. Then, inserts were separated and migrated cells were washed with PBS, observed in a microscope Leica DM IL LED (Leica Microsystems, Solms, Germany), and photographed with a Leica DFC450 C Digital Microscope Camera using Leica Application Suite software. Cells were quantified using the Cell counter complement of ImageJ (NIH, United States).

### Phagocytosis

Monocytes were cultured for 7 days in medium with 10% human serum (Musson, 1983) to assess the phagocytosis of fluorescent beads by flow cytometry and confocal microscopy. For flow cytometry, cells were seeded at 10^6^ cells/well in 6-well plates, incubated for 24 h with CM or medium and then fluorescent beads were added at 2 concentrations (10^7^ and 5 × 10^7^/ml) of FluoSpheres^®^ (Molecular Probes Thermo Fisher Scientific) and incubations proceeded for 3 h. Cells were then washed with PBS, trypsinized and resuspended in PBS to measure the fluorescence (excitation 580 nm/emission 605 nm) in a FACS-Canto II (BD Biosciences) flow cytometer. For confocal microscopy, cells were seeded at 1.2 × 10^5^ cells/well in 8-well Lab-tek microchambers (Thermo Fisher Scientific) and incubated with medium or CM and fluorescent beads as indicated above. Then, samples were washed with PBS, and incubated with anti-CD45-FITC antibody overnight at 4°C. Slides were mounted in ProLong^®^ Gold with DAPI (Molecular Probes; Invitrogen) and observed in a confocal microscope (Olympus FV1000). The percentage of phagocytosis was calculated using the number of cells with engulfed fluorescent beads and the total cell number.

### Senescence-Associated β-Galactosidase (SA-β-Gal) Assay

Monocytes were seeded at 20 × 10^3^ cells/well in Lab-tek chambers (Thermo Fisher Scientific) and incubated with CM in the presence or absence of LPS (1 μg/ml) at 37°C for 3 days. SA-β-Gal activity was measured using the cellular senescence staining kit (Cell Biolabs, San Diego, CA, United States). Cells were fixed with 0.25% glutaraldehyde in PBS for 5 min at room temperature and incubated with staining solution at 4°C overnight. Slides were mounted in Prolong Gold antifade reagent with DAPI (Molecular Probes, Invitrogen, Thermo Fisher Scientific) and examined under a microscope (Leica DM IL LED). Slides were photographed with a Leica DFC450 Digital Microscope Camera using the Leica Application Suite software.

### Oxidative Stress

Monocytes were seeded into 6-well plates at a density of 10^6^ cells/well in medium with 10% human serum and incubated until semi-confluence. The medium was then replaced by CM in treated wells or by the medium in controls. After 24 h incubation, cells were stimulated with 1 μg/ml of LPS (Sigma-Aldrich) for 30 min. After washing with medium without phenol red, a solution of dihydrorhodamine (5 μM, Sigma-Aldrich) in this medium was added and cells were incubated for 15 min at 37°C. The supernatant was then discarded, cells were washed several times with PBS and resuspended in PBS to measure the fluorescence (excitation 485 nm/emission 534 nm) in a FACS-Canto II (BD Biosciences) flow cytometer.

### Mitochondrial Transmembrane Potential

The mitochondrial transmembrane potential (Δψm) was assessed with the JC-1 probe (5,5′,6,6′-tetrachloro-1,1′,3,3′-tetraethyl-benzamidazolylcarbocyanine iodide, Thermo Fisher Scientific). This lipophilic membrane-permeant cation exhibit potential-dependent accumulation in mitochondria, indicated by a fluorescence emission shift from ∼525 nm (monomeric form) to∼590 nm (aggregated form). Monocytes were seeded into 6-well plates (10^6^ cells/well) in medium with 10% human serum and incubated until semi-confluence. The medium was then replaced by CM in treated wells or by the medium in controls. After 24 h incubation, cells were stimulated with 1 μg/ml of LPS (Sigma-Aldrich) for 30 min. Cells were trypsinized, resuspended in 1 ml of PBS, and incubated with 10 μg/ml of JC-1 dye for 10 min at 37°C and 5% CO_2_. Then, cells were washed and resuspended in PBS. Both red and green fluorescence emissions were analyzed by flow cytometry using an excitation wavelength of 488 nm and observation wavelengths of 530 nm for green fluorescence and 585 nm for red fluorescence, in a Becton Dickinson FACS-Canto II cytometer (BD Biosciences).

### NF-κB Activation

Monocytes were incubated with CM in the presence or absence of LPS (1 μg/ml) at 37°C for 20 h. Nuclear factor-κB (NF-κB) binding to DNA was quantified by ELISA in nuclear extracts using the Nuclear Extract Kit Active Motif for nuclei extraction followed by TransAM p65 NF-κB Activation Assay kits (Active Motif Europe, Rixensart, Belgium), according to the manufacturer’s recommendations.

### Macrophage Polarization

Human monocytes were isolated from buffy coats and seeded as we have described before. After 2 h incubation, the adherent cells were washed with medium and incubated for 6 days in medium with 5% human serum and 25 ng/ml of human recombinant macrophage colony stimulating factor (Promokine, Heidelberg, Germany) to obtain non-polarized macrophages Mϕ (Bertani et al., 2017). M1 polarization from Mϕ was induced by supplementation of the medium with human recombinant interferon-γ (10 ng/ml, Promokine) and LPS (100 ng/ml, Sigma-Aldrich) for 48 h. M2 polarization from Mϕ was obtained by supplementing cells with human recombinant IL-4 (20 ng/ml, Promokine) for 48 h. Mϕ, M1, and M2 macrophages were incubated in the presence or absence of CM from ASC for 24 h and cytokines were measured in supernatants by ELISA. The assay for TNFα has been indicated above. The levels of transforming growth factor β1 (TGFβ1) and IL-10 were determined by ELISA assays from eBioscience (Labclinics, Barcelona, Spain), with sensitivities of 8 and 2 pg/ml, respectively.

### Statistical Analysis

The data were analyzed by one-way analysis of variance followed by Sidak’s test using the GraphPad Prism 7.0 software (Graph Pad Software, La Jolla, CA, United States). A *p*-value of less than 0.05 was considered to be significant.

## Results

### Production of Inflammatory Mediators

**FIGURE 1:**
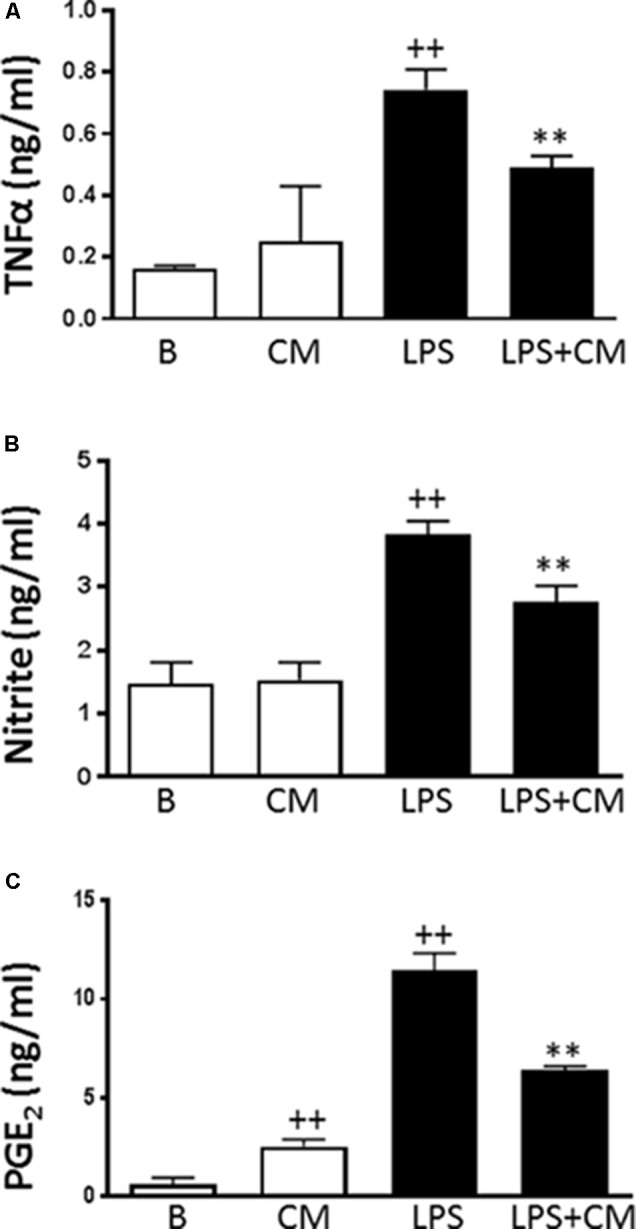
TNFα, PGE_2_ levels and NO production in human monocytes. TNFα **(A)** was measured by ELISA, nitrite levels **(B)** by fluorometry and PGE_2_ **(C)** by radioimmunoassay in cell culture supernatants. Cultures were treated with LPS alone or in combination with CM for 24 h (mean ± SD from 5 separate experiments with cells from separate donors). ++*P* < 0.01 compared to control (B: nonstimulated cells); ^∗∗^*P* < 0.01 compared to LPS.

First, we assessed the possibility of a cytotoxic effect of CM or LPS in our experimental conditions. No decrease in cell viability by the MTT method was observed after incubation of human monocytes with CM in the presence or absence of LPS for the times used in our experiments (data not shown). We then investigated the influence of CM on the production of inflammatory mediators. Cells were stimulated with LPS in the presence or absence of CM, and culture medium was sampled at 24 h to measure the accumulation of inflammatory mediators. **Figure 1** shows that LPS induced the production of TNFα. Although CM did not modify basal levels of TNFα, it significantly decreased the production of this cytokine induced by LPS (**Figure 1A**). Nitrite accumulation, an index of NO synthesis, was measured in the culture medium by a fluorometric method. As shown in **Figure 1B**, nitrite levels in nonstimulated cells were similar to those of CM. Nevertheless, we observed a 2.5-fold higher amount of nitrite in the medium after 24 h of monocyte stimulation with LPS and the levels of this mediator were significantly reduced in the presence of CM. LPS strongly induced PGE_2_ production (**Figure 1C**). Treatment of nonstimulated cells with CM enhanced the levels of PGE_2_ whereas CM significantly reduced the accumulation of this eicosanoid in the presence of LPS stimulation (**Figure 1C**).

### Myeloperoxidase Release

**FIGURE 2:**
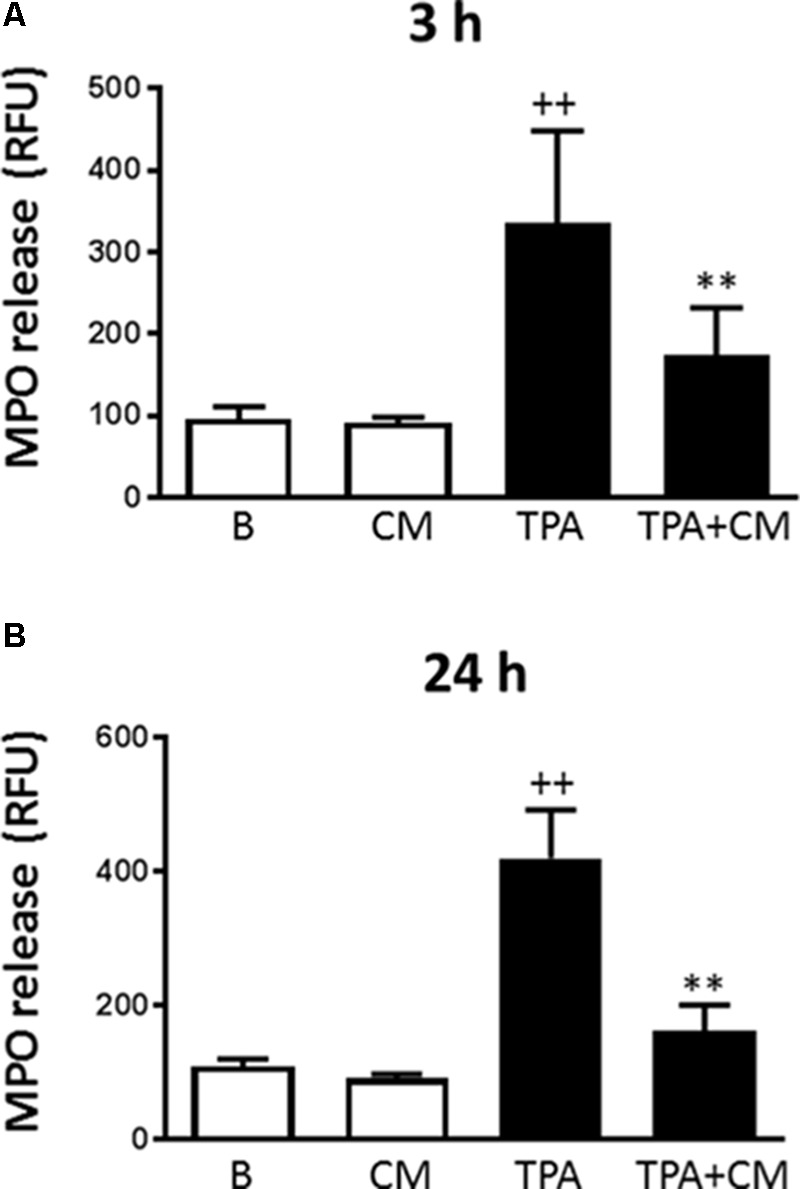
Myeloperoxidase release by human monocytes. Myeloperoxidase activity in cell culture supernatants was measured by a fluorometric procedure after 3 h **(A)** or 24 h **(B)** of TPA stimulation in the presence or absence of CM (mean ± SD from 4 separate experiments with cells from separate donors). ++*P* < 0.01 compared to control (B: nonstimulated cells); ^∗∗^*P* < 0.01 compared to TPA. RFU, relative fluorescence units.

**Figure 2A** shows that LPS induced the release of myeloperoxidase into the culture medium by 3.5-fold after 3 h of stimulation. Treatment with CM significantly decreased this process by 48%. A similar stimulation was observed after 24 h of incubation with LPS (**Figure 2B**) but CM treatment resulted in a higher reduction of myeloperoxidase release (by 61%). These results demonstrate that CM exerts inhibitory effects on monocyte degranulation.

### Cell Migration

**FIGURE 3:**
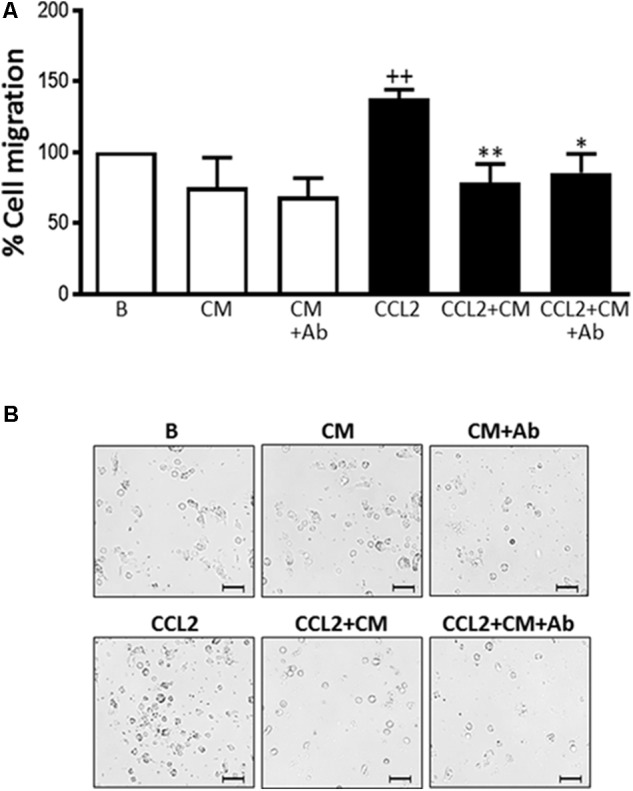
Monocyte migration induced by CCL2. Cell migration was measured after 72 h. **(A)** Quantitative analysis (mean ± SD from 4 separate experiments with cells from different donors), ++*P* < 0.01 compared to control (B: nonstimulated cells); ^∗^*P* < 0.05, ^∗∗^*P* < 0.01 compared to CCL2. **(B)** Representative images. Microscopic magnification of the objective lens 20 × . Bar = 100 μm.

The migration of human monocytes was analyzed using transwell migration chambers and the monocyte-attracting chemokine CCL-2 as the stimulus. This chemokine plays a major role in regulating the movement of myeloid cells into inflammatory sites (Hayashida et al., 2001). CCL-2 significantly promoted migration when compared to nonstimulated monocytes (**Figure 3**). Incubation in the presence of CM resulted in a significantly lower number of recruited monocytes compared with CCL-2 controls. Therefore, migration was reduced to a level below that of nonstimulated cells. To exclude a possible influence of heparan sulfate proteoglycan on the observed effects of CM, some incubations were performed after the neutralization of this proteoglycan with a specific antibody. As shown in **Figure 3**, this treatment did not modify the inhibitory effect of CM. These results indicate that CM is able to down-regulate the chemotactic response induced by CCL2 in human monocytes.

### Phagocytosis

**FIGURE 4:**
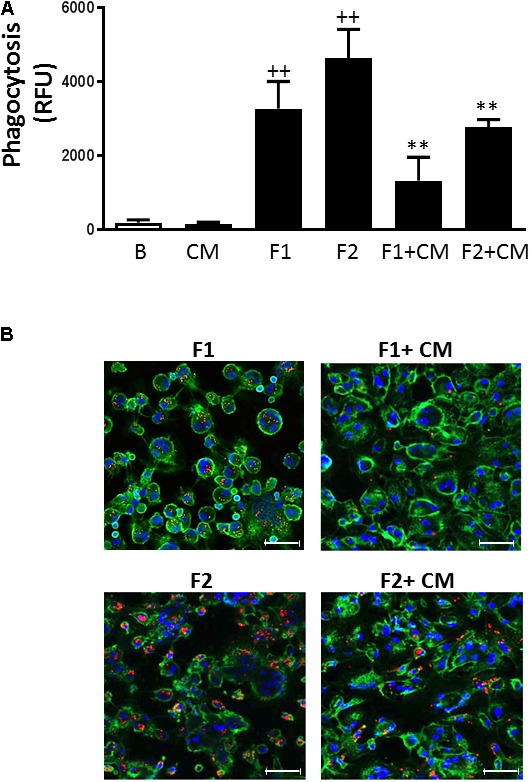
Phagocytosis in human macrophages. Phagocytosis of fluorescent beads was determined after 3 h. **(A)** Quantitative analysis (mean ± SD from 4 separate experiments with cells from different donors), ++*P* < 0.01 compared to control (B: nonstimulated cells); ^∗∗^*P* < 0.01 compared to F1 or F2, as appropriate. **(B)** Representative fluorescent images. CD45-FITC (green), DAPI (blue), fluorescent beads (red). Concentrations of fluorescent beads: F1, 10^7^/ml and F2, 5 × 10^7^/ml. Microscopic magnification of the objective lens 20 × . Bar = 50 μm.

Non-opsonic phagocytosis can trigger the release of inflammatory mediators and contribute to tissue injury (Ofek et al., 1995). As shown in **Figures 4A,B**, fluorescent beads at the concentrations used (F1: 10^7^/ml and F2: 5 × 10^7^/ml) were phagocytosed in a concentration-dependent manner by human monocytes. When cells were treated with CM we observed significant reductions in this process by 60 and 40%, respectively.

### SA-β-Gal Activity

**FIGURE 5:**
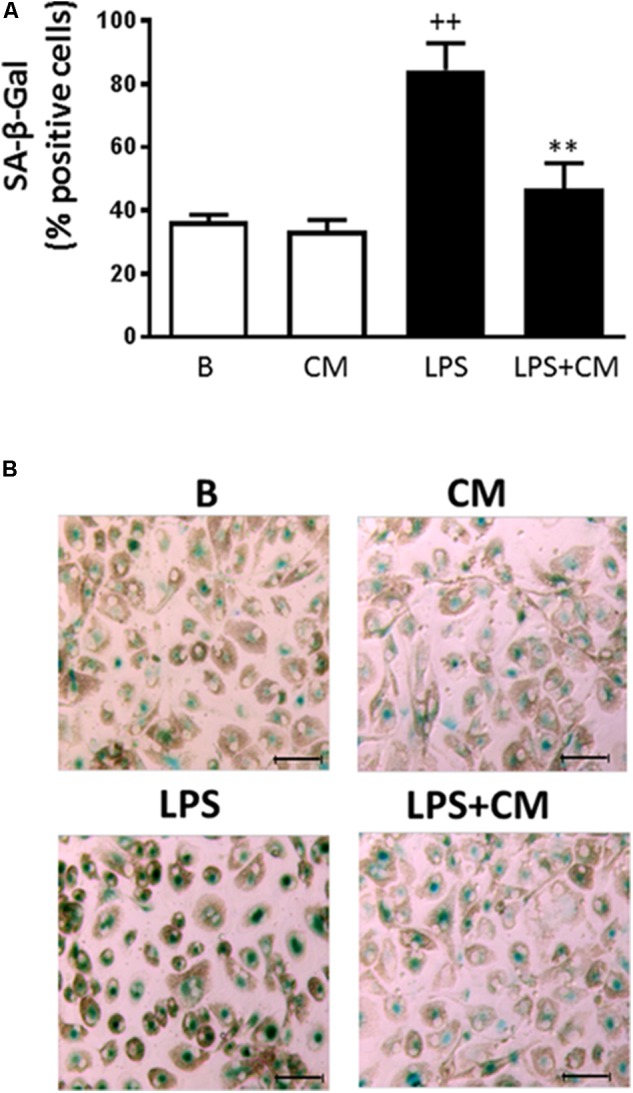
Analysis of human monocyte senescence. SA-β-Gal was determined after 72 h stimulation with LPS. **(A)** Percentage of SA-β-Gal positive cells (mean ± SD from 3 separate experiments with cells from different donors). ++*P* < 0.01 compared to control (B: nonstimulated cells); ^∗∗^*P* < 0.01 compared to LPS. **(B)** Representative microscopic fields of SA-β-Gal (green) positive cells. Microscopic magnification of the objective lens 20 × . Bar = 50 μm.

Senescent cells exhibit increased cytoplasmic activity of SA-β-Gal (Dimri et al., 1995). To examine whether CM may affect senescence, we characterized this process by the presence of SA-β-Gal–positive cells. Incubation of monocytes with LPS for 3 days induced a significant increase in the percentage of SA-β-Gal-positive cells (85%) compared with 36% in nonstimulated controls (**Figures 5A,B**). Treatment with CM induced a significant reduction (47%) of LPS effects on this marker of senescence.

### Oxidative Stress

**FIGURE 6:**
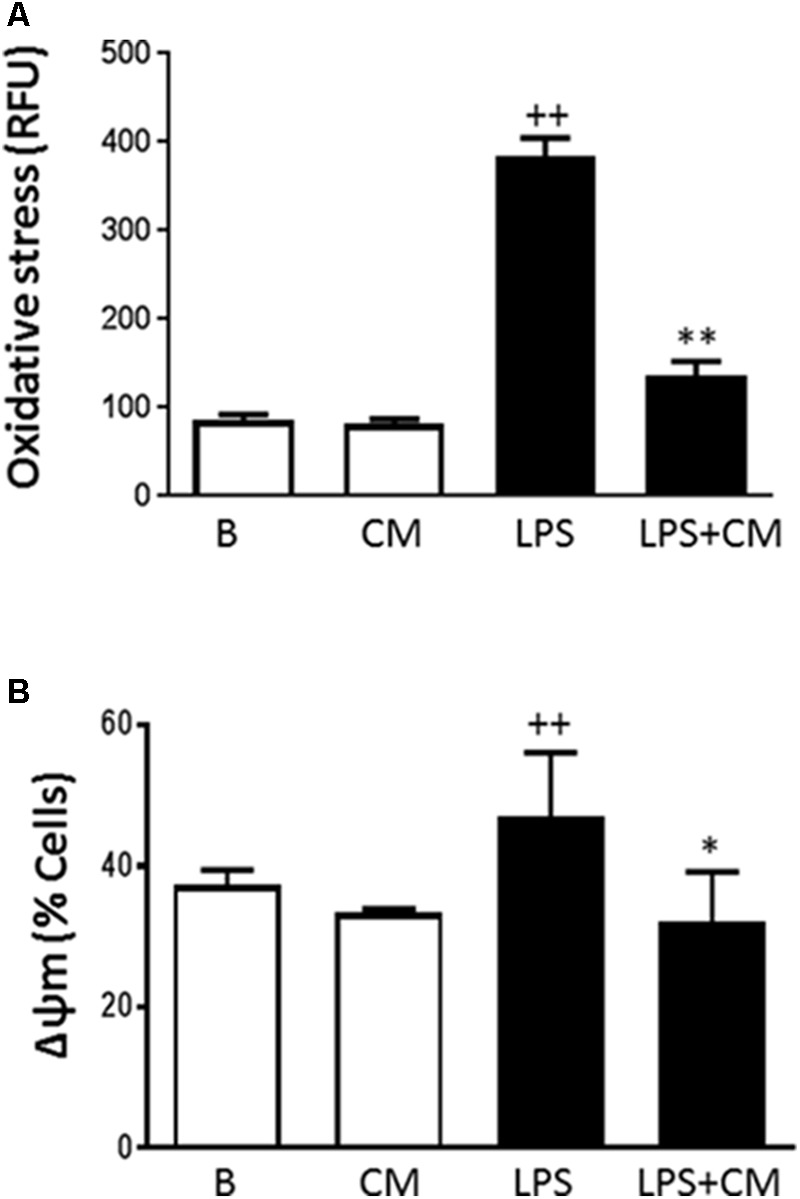
Oxidative stress **(A)** and mitochondrial membrane potential **(B)** in human monocytes. Analysis was performed by flow cytometry using dihydrorhodamine **(A)** or the probe JC-1 **(B)**. Cultures were treated with LPS alone or in combination with CM. Results are expressed as relative fluorescence units (RFU) **(A)** or percentage of cells with altered mitochondrial potential **(B)** (mean ± SD from 3 separate experiments with cells from separate donors). ++*P* < 0.01 compared to control (B: nonstimulated cells); ^∗^*P* < 0.05, ^∗∗^*P* < 0.01 compared to LPS.

Oxidative stress plays an important role in the induction of premature senescence and contributes to tissue injury in inflammation (Ben Porath and Weinberg, 2005). The change in ROS levels following exposure of human monocytes to LPS was assessed and the results are shown in **Figure 6A**. LPS stimulation of human monocytes resulted in a 4.5-fold increase in oxidative stress and this process was significantly inhibited (65%) by CM treatment.

### Mitochondrial Membrane Potential

Because we observed an inhibitory effect of CM on oxidative stress, we were interested to determine whether CM could modify the changes in mitochondrial membrane potential induced by LPS. The probe JC-1 was used to measure changes in the mitochondrial membrane potential (ΔΨ) of human monocytes. **Figure 6B** shows that LPS enhanced the number of cells with a low mitochondrial membrane potential whereas CM treatment counteracted the effects of LPS.

### NF-κB Activation

**FIGURE 7:**
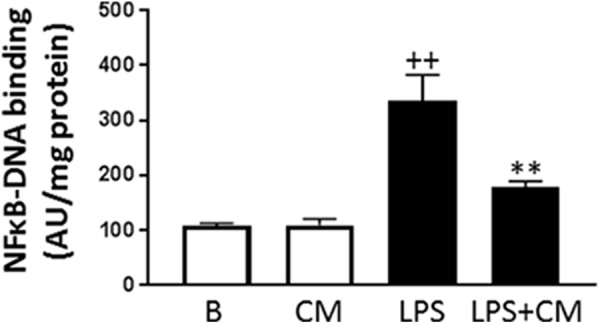
NF-κB activation. Monocytes were treated with LPS alone or in combination with CM for 20 h (mean ± SD from 3 separate experiments with cells from separate donors). ++*P* < 0.01 compared to control (B: nonstimulated cells); ^∗∗^*P* < 0.01 compared to LPS. AU, arbitrary units.

NF-κB is the main transcription factor involved in the synthesis of inflammatory mediators induced by LPS and cytokines. We have determined the influence of CM on the binding of p65 NF-κB to DNA in the nucleus of human monocytes stimulated with LPS. As shown in **Figure 7**, LPS significantly increased NF-κB binding to DNA whereas in monocytes treated with CM, we observed a significant reduction of this process.

### Macrophage Polarization

**FIGURE 8:**
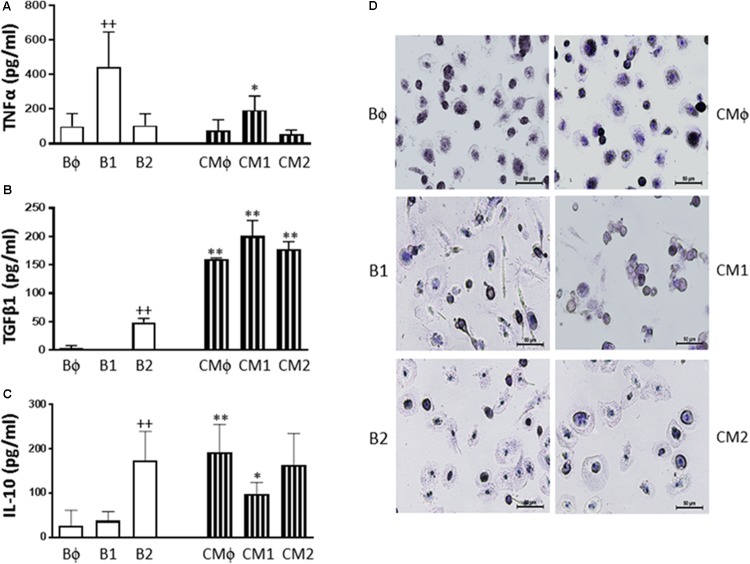
TNFα **(A)**, TGFβ1 **(B)** and IL-10 **(C)** production by non-polarized and polarized monocyte-derived macrophages, and cell morphology **(D)**. Cultures of Mϕ, M1, and M2 macrophages were treated with either culture medium (basal: Bϕ, B1, and B2, respectively) or CM (CMϕ, CM1, and CM2, respectively) for 24 h (mean ± SD from 3 separate experiments with cells from separate donors). ++*P* < 0.01 for B1 or B2 compared with Bϕ; ^∗^*P* < 0.05, ^∗∗^*P* < 0.01 for CMϕ, CM1, or CM2 compared with the corresponding basal group (Bϕ, B1, or B2). Cell morphology was observed after hematoxylin staining. Microscopic magnification of the objective lens 20 ×. Bar = 50 μm.

Non-differentiated (Mϕ) as well as classically (M1) and alternatively (M2) polarized monocyte-derived macrophages were incubated in the presence or absence of CM. As shown in **Figure 8**, Mϕ macrophages released into the medium TNFα accompanied by very low levels of TGFβ1 or IL-10. We observed that differentiation into M1 macrophages resulted in a significant enhancement of TNFα production (**Figure 8A**) whereas M2 differentiation led to significant increases in TGFβ1 (**Figure 8B**) and IL-10 (**Figure 8C**) levels. Treatment with CM inhibited TNFα release in M1 macrophages and enhanced TGFβ1 and IL-10 levels in non-differentiated and M1 macrophages. In M2 macrophages, CM significantly increased the release of TGFβ1 whereas the levels of IL-10 were not modified. As reported previously (Bertani et al., 2017), M1 macrophages presented a higher number of spindle shaped cells compared with M2 macrophages which showed a more spread morphology (**Figure 8D**). Treatment of M1 with CM modified cell morphology toward the Bϕ phenotype.

## Discussion

A wide range of factors present at the inflammatory microenvironment activate monocyte to migrate, phagocytose and generate ROS and pro-inflammatory mediators (Shi and Pamer, 2011) which exert autocrine and paracrine stimulatory effects leading to an amplification loop to perpetuate inflammation (Bardelli et al., 2012). The results presented in this paper show that CM from ASC down-regulates the activation of monocytes/macrophages induced by different types of stimuli and controls a number of relevant pro-inflammatory functions.

LPS recognition by Toll-like receptor-4 stimulates downstream signaling pathways including NF-κB and mitogen-activated protein kinases to induce the synthesis of a variety of pro-inflammatory molecules such as cytokines, PGE_2_ and NO (Akira and Takeda, 2004). In this study we report that CM attenuates the release of crucial mediators of inflammatory responses. Therefore, CM reduced the production of TNFα, a cytokine with a central role in inflammation and tissue injury which has become an important target for the development of effective therapeutic agents in rheumatoid arthritis and other chronic inflammatory conditions (Beutler, 1999). Besides, CM reduced the production of PGE_2_ stimulated by LPS. This eicosanoid exerts pro-inflammatory effects with vasodilation, oedema formation and synthesis of matrix metalloproteinases (Martel-Pelletier et al., 2003) although in some circumstances it also exhibits immunomodulatory properties (Harris et al., 2002). CM also inhibited NO production, an important mediator that may promote inflammation in mononuclear cells (Frieri, 1998). It is interesting to note that elevated serum levels of NO have been found in inflammatory arthritis patients with severe disease activity and they correlate with monocyte expression of inducible NO synthase (Pham et al., 2003). In addition, we have shown that CM decreases the DNA binding activity of NF-κB which could be an important mechanism for its anti-inflammatory effects on human monocytes. This is in line with our previous reports showing that the inhibition of this transcription factor mediates the anti-inflammatory effects of human ASC CM on osteoarthritic chondrocytes (Platas et al., 2013) and also of mouse ASC CM in the zymosan-injected mouse air pouch (Carceller et al., 2015).

Recruitment of monocytes is necessary to control infections, but it also contributes to the pathogenesis of inflammatory diseases (reviewed in Shi and Pamer, 2011). It is known the important role of monocyte infiltration in inflamed tissues where they mediate tissue injury. We have shown that CM reduces monocyte migration induced by the potent chemoattractant CCL2. Enhanced levels of this chemokine have been demonstrated in inflammatory conditions, e.g., in synovial fluid of rheumatoid arthritis patients (Akahoshi et al., 1993). CCL-2 induces a quick calcium influx and cell activation and polarization which contribute to the establishment of inflammation in concert with additional signals (Mukaida et al., 1998). We have also investigated the possible effect of CM on another cellular function, the non-opsonic phagocytosis of inert particles. Our results indicate that CM is able to modulate the phagocytic properties of human monocytes/macrophages, which may contribute to the control of the inflammatory response as phagocytic stimuli can trigger or potentiate the production of inflammatory mediators (Corradin et al., 1991).

Monocytes are activated by a variety of stimuli to produce superoxide anion in a process primarily mediated by the NADPH oxidase complex (Cathcart, 2004). ROS act as signaling molecules that regulate cell growth, adhesion, differentiation, senescence, and apoptosis. On the other hand, there are synergistic interactions between ROS and inflammatory agents to mediate the inflammatory response with promotion of endothelial dysfunction, migration of leukocytes across the endothelium and tissue injury (Mittal et al., 2014). In addition, NO and superoxide interact to form the potent oxidant peroxynitrite able to interact with lipids, DNA, and proteins leading to cell damage. Peroxynitrite may also be involved in NF-κB activation and cytokine release in human monocytes (Matata and Galinanes, 2002) and represents an important pathogenic mechanism in chronic inflammatory diseases (Pacher et al., 2007). Our data also indicate that CM reduces myeloperoxidase release by human monocytes. This observation may be relevant in relation with the control of oxidative stress as myeloperoxidase catalyzes the production of potent oxidants such as hypochlorous acid from hydrogen peroxide and chloride anion. These oxidants amplify the potency of ROS and have been implicated as mediators of oxidative tissue damage and cellular dysfunction in the development of many inflammatory conditions (Rayner et al., 2014).

ROS can induce and stabilize cell senescence, a process characterized by mitochondrial dysfunction and elevated ROS production which is related to chronic inflammatory diseases (reviewed in Correia-Melo et al., 2014). Cell senescence may contribute to the development of chronic inflammatory diseases (Mytych et al., 2017). In particular, accumulation of senescent monocytes has been associated to chronic inflammation in conditions such as atherosclerosis (Merino et al., 2011). Inflammatory and oxidative and nitrosative stress by low-dose LPS can induce premature senescence due to DNA damage, proliferation inhibition, or conversion of protective monocytes to cells showing a secretory and pro-inflammatory phenotype that may alter cell function and enhance adhesion to endothelial cells (Mytych et al., 2017). Our data indicate that CM from ASC protects human monocytes from the pro-senescence effects of LPS.

Macrophages play a key role in chronic inflammatory diseases and upon activation are a main source of TNFα in inflamed tissues. In addition, these cells release ROS, nitrogen species, PGs and matrix-degrading enzymes, and contribute to phagocytosis and antigen presentation (Haringman et al., 2005). In the presence of different pathophysiological conditions and microenvironments, macrophages can acquire distinct functional phenotypes. Classically (M1) and alternatively (M2) polarized macrophages possess pro-inflammatory and anti-inflammatory and reparative functions, respectively. Our results indicate that ASC exert paracrine actions on differentiated monocyte-derived macrophages to potentiate anti-inflammatory cytokines markers of M2 macrophages while simultaneously reducing the pro-inflammatory cytokine TNFα marker of M1 macrophages. Therefore, ASC may down-regulate the inflammatory response and favor the development of homeostasis and repair processes. These findings are in line with reports of M2 macrophage polarization by ASC, a property responsible for accelerated wound healing in animal models (Zheng et al., 2015).

The secretome of ASC has a complex composition including soluble factors and microparticles (Tofiño-Vian et al., 2018). Although some molecules such as IL-10 may contribute to the anti-inflammatory properties of ASC CM, recent data suggest a more relevant role for extracellular vesicles. In fact, we have demonstrated that microvesicles and exosomes from ASC CM are the main anti-inflammatory mediators in human osteoarthritic osteoblasts (Tofiño-Vian et al., 2017). We are performing further studies to know the complex mechanisms involved in the regulation of the inflammatory process by the ASC secretome. The results of our study suggest that ASC paracrine actions may determine the evolution of inflammation by modulating the functions of monocytes/macrophages which play an important role in the innate immune response.

## Author Contributions

MIG and MJA participated in the design of research. JP, MDPdC, and VM performed the experiments. MIG, JP, and MJA performed the data analyses. All authors approved the final version of the manuscript and agree to be accountable for all aspects of the work ensuring that questions related to the accuracy or integrity of any part of the work are appropriately investigated.

## Conflict of Interest Statement

The authors declare that the research was conducted in the absence of any commercial or financial relationships that could be construed as a potential conflict of interest.
